# Still Higher Risk for Burnout and Low Work Engagement Among Female Residents After 10 Years of Demographic Feminisation

**DOI:** 10.1007/s40670-024-02084-y

**Published:** 2024-06-18

**Authors:** Maud Kramer, Karen D. Könings, Jelle T. Prins, Frank M. M. A. van der Heijden, Ide C. Heyligers

**Affiliations:** 1 School of Health Professions Education, Faculty of Health, Medicine and Life Sciences, Maastricht University, Universiteitssingel 60, 6229 ER Maastricht, Netherlands; 2 Department of Orthopedic Surgery and Traumatology, Zuyderland Medical Centre, Heerlen/Geleen, Netherlands; 3 University Medical Centre Groningen, Groningen, Netherlands; 4 Vincent van Gogh Institute for Psychiatry, Venray, Netherlands

**Keywords:** Burnout, Work engagement, Medical residents, Gender differences, Feminisation

## Abstract

**Objectives:**

We explored whether gender differences in burnout and work engagement characteristics among residents changed after the representation of female physicians has surpassed the 30% threshold of critical mass between 2005 and 2015, as well as if these gender differences are influenced by working in a surgical versus a non-surgical specialty.

**Methods:**

This study used data of two questionnaire surveys on the well-being of Dutch residents, collected in 2005 (*N* = 2115) and 2015 (*N* = 1231). Burnout was measured with the validated Dutch translation of the Maslach Burnout Inventory, covering the characteristics emotional exhaustion, depersonalisation and personal accomplishment. Work engagement was measured with the Utrecht Work Engagement Scale, covering the characteristics vigour, dedication and absorption. Gender differences in residents’ engagement and burnout characteristics in 2005 and 2015 were analysed using hierarchical regression analyses. Factorial analyses of variance were used to compare gender differences in residents’ burnout and engagement characteristics in general surgery with those in internal medicine.

**Results:**

In both years, female residents reported higher emotional exhaustion, lower depersonalisation, personal accomplishment, and vigour than males. These gender differences were similar in general surgery and internal medicine.

**Conclusions:**

This study demonstrated unchanged gender differences in burnout and work engagement characteristics among residents after 10 years of demographic feminisation (increasing female representation), indicating higher risk for burnout and lower work engagement among females, both in surgical and non-surgical specialties. In view of the ever-increasing number of female residents, educators and hospitals need to create supporting work environments that safeguard residents’ well-being.

## Introduction

Burnout in medical residents is highly prevalent, with burnout rates described up to 50%, varying by medical specialty [1, 2]. Earlier studies found that the risk for burnout was higher in female doctors than in their male colleagues, both among residents and among physicians, particularly in surgical specialties [2–6]. Prins et al. found that female residents experienced lower work engagement [7]. These findings of low work engagement and high risk for burnout in female residents are alarming, since there has been a world-wide increase in the representation of women in the medical profession, also called demographic feminisation [8].

Low well-being can undermine residents’ professional development, place residents’ physical health at risk and negatively influence the quality of patient care [3]. Burnout and work engagement are two important indicators for measuring employees’ well-being [9]. Burnout is characterized by high levels of emotional exhaustion and depersonalisation (feelings of cynicism and detachment toward patients and colleagues) and low levels of personal accomplishment (feelings of efficacy) [9]. As a result of gender differences in coping with stress, burnout characteristics seem to be gendered [10]. Men report more depersonalisation compared to women, while women report more emotional exhaustion compared to men [10, 11].

As a counterpart of burnout, work engagement is a positive and fulfilling work-related state of mind [7, 12]. Engagement can be a protective factor against burnout and is characterized by high levels of vigour (energy and willingness to invest in work), dedication (feelings of pride, enthusiasm, and inspiration in work) and absorption (being fully concentrated and happily engrossed in work) [12, 13]. According to Maslach et al., burnout can result from chronic feelings of misfit between individuals and their work environment, while engagement arises from feelings of fit between individuals and their work environment [9].

Several sociocultural and ﻿anthropological studies described the stereotypical masculine work culture of general surgery [14–17]. Within the surgical field, masculine norms and values make it difficult for women to successfully position themselves as female individuals [15–17]. As physicians in surgical specialties are still predominately male, these specialties also lack women as role models with whom female residents can identify [18, 19]. Moreover, female surgical doctors experience high levels of gender bias in their work environment, which negatively impacts their well-being [18, 20–22].

The critical mass theory proposed in social studies in the fields of politics and sales states that a critical mass of 30 to 35% female representation can cause evident changes in work culture, creating a more female-friendly work environment [23, 24]. From 2005 to 2015, the female representation among Dutch registered physicians, the educators and role models in postgraduate medical training (PGMT) increased from 27 to 40% (Appendix 1). Referring to the critical mass theory, this development might have positively influenced the work environment for Dutch female residents, resulting in stronger feelings of fit between themselves and their work environment and leading to less burnout and more work engagement. However, despite the representation of female physicians has surpassed the 30% threshold of critical mass between 2005 and 2015, the representation of female physicians in leadership positions falls behind [25]. For example, the percentage of Dutch female professors in academic hospitals, who are often head of departments and closely involved in PGMT, was still 19% in 2015 and first reached 30% in 2023 [26]. Some researchers suggest that mainly approaching the critical mass threshold of female leaders is necessary for realizing cultural change [27].

No earlier study directly assessed potential changes in the burnout and work engagement characteristics of female and male residents after a period of time in where the representation of female physicians has surpassed the 30% threshold of critical mass. Besides, as earlier studies show a negative influence of the surgical work-environment mainly on the well-being among female residents [4, 5, 20], it seems important to directly compare gender differences among residents from surgical versus non-surgical specialties. In view of the ever-increasing number of female residents worldwide [8], there is a need to explore potential changes in gender differences in burnout and work engagement among residents over time and between specialties, so PGMT programs can be adapted to resident’s needs.

The aim of this study was to explore whether gender differences in burnout and work engagement characteristics among Dutch residents from various medical specialties changed after the 10-year period from 2005 to 2015 in where the representation of female physicians has surpassed the 30% threshold of critical mass. Furthermore, we aimed to compare gender differences in burnout and engagement characteristics among residents in general surgery, the largest surgical specialty, versus internal medicine, and the largest non-surgical specialty (Appendix 1).

## Methods

### Design and Participants

Cross-sectional national data were retrieved from the self-reported questionnaires of two surveys among Dutch medical residents, one of which took place in October 2005 and the other in September 2015. In 2005, the survey was coordinated by the Dutch Medical Registration Committee. All 5245 residents who were registered by the Dutch Medical Registration Committee as being in PGMT at the moment of sampling were invited. In 2015, the survey was no longer coordinated by the Dutch Medical Registration Committee, but by the Dutch Junior Doctor Association. Only residents who were members of the Dutch Junior Doctor Association were invited, being at the moment of sampling 2519 residents (36.4%) of all registered Dutch residents (7141). In the invitation e-mail, members of the association were encouraged to share the link for the questionnaire with other, non-member residents.

In both years, an information letter stated the goal of the study as investigating well-being in Dutch residents. Also, in both years, three reminders were sent within a period of two months. At the time of data collection, ethical approval by the ethics review board was not required under Dutch law. Following the current guidelines of the national Research Ethics Committee of the Netherlands Association for Medical Education and in accordance with the Declaration of Helsinki, participation in the present study was voluntary, all participants provided written informed consent, and data were analysed anonymously.

There was no overlap between the respondents of the questionnaires issued in 2005 and in 2015, since participants’ self-reported maximum “years in training” at the moment of sampling in 2015 was 9 years.

### Measures

Besides burnout and work engagement characteristics, respondents were asked about their gender, age, medical specialty, number of years in training at the time of sampling, and clinical setting of PGMT at the moment of sampling (university medical hospital, general teaching hospital, mental health clinic, or other types of institute).

#### Burnout

Burnout was measured using the validated Dutch translated version of the Maslach Burnout Inventory Human Services Survey (MBI-HSS): the Utrecht Burnout Scale for contact-based professions (UBOS-C) [28, 29]*.* Like the MBI-HSS, the UBOS-C consists of 20 items covering three domains of burnout: emotional exhaustion (8 items), depersonalisation (5 items), and personal accomplishment (7 items) [28]. Items were rated on a 7-point Likert scale, ranging from 0 (never) to 6 (always/daily). Different to the MBI, but in line with the UBOS manual, we computed means per subscale (instead of sum scores, which are mostly used for MBI) ranging from 0 and 6. In line with recent recommendations of the 4th edition of the MBI manual and of Rotenstein et al., we exclusively analysed burnout characteristics as continuous variables and did not use cut-off scores for subscales nor combined subscales to analyse burnout as a syndrome [29, 30]. High scores on emotional exhaustion and depersonalisation and low scores on personal accomplishment denote higher risk for burnout. As men from all work fields score higher for the subscale depersonalisation, the UBOS manual uses different cut-off values for men and women on this subscale [28]. We take this into account, when interpreting our results on the subscale depersonalisation of female and male participants.

#### Work Engagement

Work engagement was measured using the Dutch Utrecht Work Engagement Scale (UWES), which comprises nine items divided into three subscales: vigour (three items), dedication (three items), and absorption (three items) [13]. Items were rated on a 7-point Likert scale from 0 to 6, with the same labels as in the UBOS. Similar to the UBOS, mean scores range between 0 and 6. Higher mean scores per subscale denote higher engagement.

### Analyses

Data were analysed in Jamovi 0.9.0.3 [31]. To confirm the grouping of items in the UBOS and UWES as published earlier, we performed confirmatory factor analysis for each year of data collection (2005 and 2015), with full information maximum likelihood estimation for missing responses [32].

To calculate gender differences between residents’ burnout and work engagement characteristics in 2005 and 2015, we performed two-level hierarchical regression analyses to account for respondents being nested within specialties: data of respondents within a certain specialty were expected to be more strongly correlated with each other than data of respondents from different specialties [33]. First, we computed basic models with the variables year and gender per subscale of the UBOS and the UWES to test gender differences in residents’ burnout and work engagement characteristics while adjusting for year of data collection (2005 or 2015). Second, we added the “gender × year” interaction variable to the models to examine if gender differences in residents’ burnout and engagement characteristics differed between the years.

Besides, we compared gender differences between the burnout and engagement characteristics of residents in general surgery and those in internal medicine by means of factorial analyses of variance (ANOVA) per subscale of the UBOS and UWES. All ANOVA models included the variables year, gender, specialty (in these models as a binary variable: general surgery or internal medicine) and the “gender × specialty” interaction. To correct for multiple testing, effects with *p* < 0.01 were considered statistically significant.

## Results

### Response Rates and Demographic Variables

In 2005, the national response rate was 41% (2115 out of 5140 nationally registered residents). In 2015, 1231 residents participated in the questionnaire, representing 17.2% (1231 out of 7141) of the nationally registered population of residents. However, due to the sampling procedure used in 2015, an exact response rate could not be calculated for this year. Eight of the 2115 participants in 2005 and 83 of the 1231 participants in 2015 were excluded from the analyses because they did not report their gender or medical specialty.

In 2015, the percentage of female participants was 74% (830/1148), which was significantly higher than in 2005, when it was 61% (1289/ 2107) (*X*^2^ (1) = 53.59, *p* < 0.001). The numbers and percentages of female and male residents per medical specialty who completed the questionnaire in 2005 and 2015 are presented in Appendix 2. In both years, the mean age of the participants was 32 years (31.51 years; SD 3.54 in 2005, and 31.57 years; SD 3.64 in 2015). Residents had been in PGMT for an average of 3 years (3.08 years; SD 1.49 in 2005, and 3.15 years; SD 1.57 in 2015) with a range from first year (or “just started”) in residency to 9 years in residency. The length of residency training programs in the Netherlands varies between 4 and 6 years full-time. During their training years, residents rotate between different hospitals. At the moment of sampling in 2005 and in 2015, 48%_(2005)_ versus 44%_(2015)_ of the residents were in training at a university hospital, 41%_(2005)_ versus 44%_(2015)_ at a general teaching hospital, 9%_(2005)_ versus 6%_(2015)_ at a mental health clinic and 2%_(2005)_ versus 1%_(2015)_ at a rehabilitation centre. Table 1 shows the descriptive statistics of the UBOS and UWES for female and male residents in 2005 and 2015.

**Table 1:** Means and standard deviations of the subscales of the UWES and UBOS, separately for male and female residents in 2005 and 2015

	**UWES**			**UBOS**		
	**Vigour**	**Dedication**	**Absorption**	**Emotional exhaustion**	**Depersonalisation**	**Personal accomplishment**
**2005, male (*n* = 818)**	4.00 (1.03)	4.43 (1.03)	3.52 (1.18)	1.83 (1.04)	1.55 (0.94)	4.46 (0.76)
**2005, female (*n* = 1289)**	3.88 (1.00)	4.39 (0.97)	3.52 (1.08)	2.09 (1.05)	1.35 (0.83)	4.42 (0.72)
**2015, male (*n* = 318)**	4.06 (1.07)	4.58 (1.04)	3.87 (1.13)	1.71 (1.03)	1.32 (0.87)	4.54 (0.80)
**2015, female (*n* = 830)**	3.91 (1.06)	4.51 (1.01)	3.78 (1.10)	1.92 (0.99)	1.14 (0.79)	4.46 (0.77)

### Reliability of Measures

Confirmatory factor analyses confirmed the grouping of items in the UBOS and UWES as published earlier in their manuals [13, 28]. The outcomes are presented in Appendix 3.

### Gender Differences in Residents’ Burnout and Engagement Characteristics

In the two-level basic models (Table 2), which tested gender differences in residents’ burnout and work engagement characteristics in the total study population while adjusting for year of data collection and specialty, the following gender differences were found: female residents scored significantly higher on emotional exhaustion (*β* = .110, 95% CI [.072, .149], *p* < .001) and lower on depersonalisation (*β* =  − .070, 95% CI [− .102, − .038], *p* < .001), personal accomplishment (*β* = -.037, 95% CI [− .065, − .009], *p* = .009) and vigour (*β* =  − .052, 95% CI [− .091, − .014], *p* = 0.008) than their male peers. These two-level basic models (Table 2) also showed that all residents in 2015 scored lower on emotional exhaustion (*β* =  − .066, 95% CI [− .103, − .028], *p* < 0.001) and depersonalisation (*β* =  − .091, 95% CI [− .122, − .060]), *p* < 0.001), while they scored higher on personal accomplishment (*β* = .041, 95% CI [.014, .068], *p* = 0.003), dedication (*β* = .054, 95% CI [.017; .090], *p* = 0.004) and absorption (*β* = .131, 95% CI [.091, .172], *p* < 0.001) than their colleagues in 2005. In the two-level interaction models (Table 2), which tested if the gender differences in residents’ burnout and work engagement characteristics changed over time, no interaction effects were found between gender and year of data collection, meaning that gender differences remained unchanged between 2005 and 2015.

**Table 2:** Gender differences in residents’ work engagement and burnout characteristics in 2005 (*N* = 2107) and 2015 (*N* = 1148)

	**UWES**							**UBOS**			
	**Vigour**		**Dedication**			**Absorption**		**Emotional exhaustion**		**Depersonalisation**	**Personal accomplishment**
**β (95%CI) of two-level regression interaction models**											
Year of data collection		.010 (− .031; .050)		.057* (.018; .096)		.139* (.095; .183)		− .061* (− .101; − .021)		− .094* (− .101; − .021)	.044* (.015; .073)
Gender		− .054* (− .095; − .013)		− .025 (− .065; .015)		− .013 (− .055; .034)		.106* (.065; .147)		− .067* (− .101; − .033)	− .040* (− .069; − .010)
*Gender* × *year*		− .004 (− .043; .035)		− .008 (− .046; .030)		− .020 (− .062; .023)		− .012 (− .052; .027)		.008 (− .024; .041)	− .008 (− .035; .020)
**β (95%CI) of two-level regression basic models**											
Year of data collection		.008 (− .029; .045)			.054* (.017; .090)	.131* (.091; .172)		− .066 * (− .103; − .028)		− .091* (− .122; − .066	.041* (.014; .068)
Gender		− .052* (− .091; − .014)			− .022 (− .059; .015)	− .003 (− .045; .039)		.110* (.072; .149)		− .070* (− .102; − .038)	− .037* (− .065; − .009)
**Variance explained by the basic models**											
ICC		.025			.037		.029		.023	.079	.188
Marginal *R*^2^		.002			.003		.013		.012	.018	.004
Conditional *R*^2^		.027			.040		.041		.035	.095	.192

Intraclass coefficient (ICC) = random intercept variance / (random intercept variance + residual variance). Marginal *R*^2^ is for fixed effects; conditional *R*^2^ includes the random effects

Given the non-significant outcomes of the interaction models, we reported the ICC, marginal *R*^2^ and conditional *R*^2^ for the basic models only

^*^Statistically significant effect, *p* < .01

### Comparing Gender Differences in General Surgery and in Internal Medicine

In the ANOVA models (Table 3), which compared gender differences between the burnout and work engagement characteristics of residents in general surgery (*N*_2005_ = 170; *N*_2015_ = 71) and internal medicine (*N*_2005_ = 291; *N*_2015_ = 104), no significant interaction was found between gender and specialty, meaning that gender differences in burnout and work engagement characteristics did not differ between the two specialties. When adjusted for year and gender, residents in general surgery scored significantly higher on all three subscales of the UWES (vigour, *F* (1,628) = 32.03, *p* < 0.001; dedication *F* (1,627) = 34.23, *p* < 0.001; and absorption *F* (1,621) = 33.25, *p* < 0.001) and lower on emotional exhaustion (*F* (1,626) = 21.64, *p* < 0.001) than their colleagues in internal medicine.

**Table 3:** Comparing gender differences in residents’ work engagement and burnout characteristics between general surgery and internal medicine

	**UWES**			**UBOS**		
	**Vigour**	**Dedication**	**Absorption**	**Emotional exhaustion**	**Depersonalisation**	**Personal accomplishment**
**Mean (standard deviation)**						
Male, surgery (*n* = 142)	4.44 (0.97)	4.88 (0.93)	4.14 (1.11)	1.42 (0.93)	1.35 (0.87)	4.73 (0.73)
Male, internal medicine (*n* = 122)	3.81 (0.91)	4.18 (1.01)	3.34 (1.08)	1.93 (0.97)	1.49 (0.90)	4.41 (0.69)
Female, surgery (*n* = 99)	4.13 (0.92)	4.61 (0.93)	3.89 (1.00)	1.84 (0.98)	1.45 (0.90)	4.55 (0.63)
Female, internal medicine (*n* = 273)	3.81 (1.01)	4.31 (0.99)	3.59 (1.09)	2.12 (0.99)	1.40 (0.82)	4.46 (0.68)
***F* (df)**	***F* (1,628) = **	***F* (1,627) = **	***F* (1,621) = **	***F* (,1626) = **	***F* (1,627) = **	***F* (1,620) = **
Year of data collection	1.11	3.94	6.98^^^	4.30^^^	12.80	2.41
Gender	3.76	1.06	0.07	14.98	0.14	1.36
Specialty	32.03*	34.23*	33.25*	21.64*	0.13	11.36*
Gender-by-specialty	3.24	5.14	5.83	1.42	0.89	3.26

Descriptives and factorial analyses of variance

Specialty = binary variable: general surgery versus internal medicine

^*^Statistical significant effect, *p* < .01

## Discussion

To our knowledge, this is the first study that explored changes in gender differences in burnout and work engagement characteristics among residents from various medical specialties after a 10-year period of demographic feminisation in where the representation of female physicians has surpassed the 30% threshold of critical mass. Additionally, we compared gender differences in burnout and work engagement characteristics among residents working in a surgical versus a non-surgical specialty. In general, we found that burnout and work engagement characteristics positively changed for residents of both genders during the 10-year study period. Nevertheless, gender differences in burnout and work engagement characteristics stayed unchanged, indicating higher risk for burnout and lower work engagement among female residents. Moreover, gender differences in burnout and work engagement characteristics did not differ between general surgery and internal medicine.

Regarding gender differences in work engagement, we found that female residents overall reported lower work engagement than male residents, as they reported less vigour and similar dedication and absorption. As engagement is described as a protective factor for burnout, the present finding of female residents’ lower engagement suggests that they have a higher risk to experience burnout symptoms [7].

Regarding gender differences in burnout characteristics, we found higher depersonalisation in male residents compared to female residents. This is in line with earlier studies in residents [7, 11] and with the UBOS manual which uses higher cut-off scores for men than women on the subscale depersonalisation [28]. However, overall, female residents reported more burnout characteristics than their male colleagues, as their scores on emotional exhaustion were higher and their scores on personal accomplishment were lower.

Earlier studies showed that gender differences in burnout characteristics can be partly explained by gender differences in coping with stress [10]. Males more frequently use problem-focused coping strategies, such as avoidance or detachment [11]. While detachment leads to higher levels of depersonalisation, some detachment and even a certain level of depersonalisation can be useful in preventing emotional exhaustion and burnout [11]. Female residents more frequently use emotion-focused strategies and self-blame, which leads to higher levels of emotional exhaustion and burnout [10]. From all burnout characteristics, emotional exhaustion is strongest related to burnout and to other adverse outcomes as depression or leaving PGMT [34]. On the other hand, personal accomplishment is believed to function as a buffer for work-related distress [35]. Consequently, female residents’ lower sense of personal accomplishment and higher emotional exhaustion, as found in the present study, might increase their risk for burnout.

Our findings of unchanged gender differences in burnout and work engagement among residents between 2005 and 2015 indicate that even though the representation of female physicians has surpassed the 30% threshold of critical mass, this did not positively influence well-being among female residents. Although female physicians’ representation in the Netherlands surpassed the critical mass of 30% during our study period from 2005 to 2015, female physicians’ representation in leadership positions still falls behind. Most recent numbers (2023) show that the critical mass of 30% female representation for full professor positions in academic hospitals is finally reached in the Netherlands [26]. However, with only 1.0 percentage increase, we saw the lowest growth of female professors in the past 5 years. Moreover, female representation of executive boards in Dutch academic hospitals decreased last year from 40% in 2022 to 34% in 2023 [26]. These recent numbers confirm that the current medical work culture might still mainly consist of masculine sociocultural norms and policies, which makes it hard for women to move up to and retain leadership positions [27, 36, 37]. To change the work culture in the medical field, not merely female physicians’ representation, but hospitals and their leaders, both women and men, have to stimulate and promote female physicians in leadership positions [27, 36, 38].

Although we did not directly investigate the work environment in the current study, our results of higher burnout and lower work engagement in female residents might support assumptions that the current work-environment in PGMT is not yet adequately oriented to the needs of female residents [38]. Verweij et al. demonstrated that only male residents experience protective factors for burnout in their work environment [39]. On the other hand, female residents and physicians experience stronger tensions between work and home than males [40]. Work-home tensions among female residents might originate from unconsciously hold stereotyped gender role perceptions that women should care for the family [41]. Such stereotyped gender perceptions can function as a stressor for female residents and actually decrease their physiological health [20].

Although gender differences in burnout and work engagement characteristics stayed unchanged over time, all burnout characteristics developed in the positive way (decrease in emotional exhaustion and depersonalisation, while increase in personal accomplishment), and two out of three work engagement characteristics developed in the positive way (increase in dedication and absorption) by 2015 in residents of both genders. Apparently, changed factors in the work environment had a positive influence on both male and female residents. The overall well-being of the residents in our study may have been positively influenced over time by the fact that within the timeframe of our study, all medical specialties within the Netherlands introduced competency-based PGMT [42]. During the introduction of competency-based learning, higher attention was paid to the quality of postgraduate medical education programs in the Netherlands. Reorganizing PGMT to competency-based learning improved the quality of the learning environment for residents, especially in terms of supervision and coaching and in terms of assessment [43]. Research showed that a high qualitative learning environment can be a protective factor for burnout [44].

Gender differences in residents’ burnout and engagement characteristics did not differ between general surgery and internal medicine. In other words, we found no negative influence of a surgical work environment nor a positive influence of a non-surgical work environment on the well-being of female residents. Also surprisingly, both male and female residents in general surgery scored lower on burnout and higher on engagement than male and female non-surgical residents. These findings seem inconsistent with the extensive literature that explicitly described low well-being and a relatively high risk of burnout and attrition in general surgery, especially in female surgical residents [4, 5, 45]. As most studies that reported low well-being in surgical residents were based on data from the USA, more research is needed to investigate the well-being of male and female residents in different countries, as social context and policies differ per continent and country [37, 46].

In view of the ever-increasing number of female residents in PGMT in all medical specialties, our findings of unchanged lower work engagement and higher risk for burnout among female residents over time have practical implications for adapting PGMT programs. Periodically validated burnout surveys (like the MBI) can create burnout awareness among residents and their educators [47]. Moreover, peer supporting interventions, like organized peer coach meetings, seem to have a valuable effect on residents’ well-being [47]. Such interventions should be easily available for residents during residency training.

Fundamental in this regard is that educators in PGMT and their organization need to stimulate a culture in which an open dialogue and promotion of residents’ well-being and resilience are spearheads of policy [48, 49]. Educators in PGMT should be aware of stereotyped gender role perceptions regarding family and career, especially in their female residents [41]. A supporting work environment, in particular supportive supervisors, and a highly flexible organization (e.g. flexible work schedules) can diminish work-home tensions or even convert these tensions into career facilitators, allowing work and family roles to mutually enrich each other, thereby increasing well-being, physical health and work performance [50, 51].

Mainly in surgical specialties, the representation of female residents and physicians is still relatively low. On the other hand, recent studies show the importance of gender diversity in surgical specialties, as female surgeons seem to have better patient outcomes compared to male surgeons [52]. Mentorship of female residents by female surgeons can be valuable for residents in order to reflect on institutional barriers and to clarify their professional and personal goals [38, 48]. This support will foster female residents’ “resilience”, an indicator for psychological maturity which reduces the risk for burnout and attrition [38, 48, 49]. Moreover, sponsoring of female residents by both female and male leaders in the field can help to achieve gender equity and to start quantifiable culture changes [53].

Our study has several limitations. Recruitment and distribution methods in 2005 and 2015 differed as the data sampling was done by two separate national survey projects. The response rate in 2005 was moderate and the exact response rate in 2015 is unknown as only members of the Dutch Junior Doctor Association were invited. This might have led to selection bias, as responders might differ from non-responders in their experiences of burnout and work engagement characteristics. However, earlier studies using the same database investigated the demographic differences between responders and non-responders and found no statistically significant differences in gender, age and specialty between the respondents and non-respondents in 2005 and only a slightly higher percentage of female gender in the responders’ group in 2015, while age and specialty did not differ between responders and non-responders in this year either [7, 54]. Considering the current discussion about “gender” or “sex”, it might be a limitation that we did not explicitly ask respondents if they were transgender or gender neutral. As we used a self-reported questionnaire, we assumed that participants reported their “gender”. Furthermore, burnout and work engagement are complex and multifactorial concepts that are influenced by many factors in and outside the work environment. The broader influence of the sociocultural context was beyond the scope of our study. Additionally, as data for this study were collected in 2005 and 2015, absolute values of burnout and work engagement characteristics may have been changed over the past eight years, especially due to COVID-19 pandemic effects [55]. Finally, earlier studies show that burnout prevalence peaks during residency training, while it decreases afterwards in early physicians [56]. In the course of residency training, residents seem to develop resilience, which helps them to cope with stress and theoretically can be a protective factor for burnout [49]. Therefore, well-being might increase with years and/or with clinical experience and care must be taken to extrapolate our results to long-term or to physicians.

We recommend future qualitative studies on work environment factors in relation to well-being characteristics among male and female residents within different medical specialties. Furthermore, we recommend that international studies compare gender differences in residents’ burnout and work engagement characteristics in relation to geographic contexts and social policies. Lastly, future studies should evaluate the effect of supporting interventions, e.g. the introduction of mentor programs or peer groups in PGMT programs, on burnout and work engagement characteristics in female residents.

## Conclusions

This study demonstrated unchanged gender differences in burnout and work engagement characteristics among residents after a 10-year period of demographic feminisation, indicating higher risk for burnout and lower work engagement among female residents from various medical specialties. Apparently, even though the representation of female physicians—the educators and role models in PGMT—surpassed the 30% threshold of critical mass between 2005 and 2015, this did not positively influence well-being among female residents. Furthermore, gender differences in burnout and work engagement characteristics did not differ between residents working in a surgical specialty (general surgery) to residents working in a non-surgical specialty (internal medicine). In view of the ever-increasing number of female residents within all medical specialties, the implication for educators of PGMT programs, but also for hospitals and their leaders, is to create supporting work environments in which promotion of well-being and resilience of their residents are spearheads of policy.

## Data Availability

The raw data that support the findings of this study are available from the Dutch Junior Doctor Association, but restrictions apply to the availability of these data, which were used under license for the current study, and so are not publicly available. Data are however available from the authors upon reasonable request and with permission of the Dutch Junior Doctor Association.

## Appendix 1

Nationwide numbers and percentage of registered female physicians and residents in the Netherlands in 2005 and 2015 as registered by the Dutch Medical Registration Committee.

**Table:**

**Specialty**	**Physicians’ female representation**		**Residents’ female representation**	
	**Female/total (%) 2005**	**Female/total (%) 2015**	**Female/total (%) 2005**	**Female/total (%) 2015**
Anaesthetics	320/1283 (24.9%)	605/1824 (33.2%)	136/327 (41.6%)	310/504 (61.5%)
Cardiology	87/750 (11.6%)	233/1094 (21.3%)	90/326 (27.6%)	146/371 (39.4%)
Cardiothoracic surgery	12/115 (10.4%)	15/147 (10.2%)	5/29 (17.2%)	9/32 (28.1%)
Dermatology	138/400 (34.5%)	275/555 (49.5%)	70/103 (68.0%)	138/182 (75.8%)
General surgery	117/1115 (10.5%)	300/1389 (21.6%)	133/423 (31.4%)	168/410 (41.0%)
Internal medicine	483/1793 (26.9%)	921/ 2176 (42.3%)	413/689 (59.9%)	721/985 (73.2%)
Otorhinolaryngology	60/456 (13.2%)	135/524 (25.8%)	38/94 (40.4%)	75/132 (56.8%)
Paediatrics	606/1215 (49.9%)	952/1517 (62.8%)	269/364 (73.9%)	264/332 (79.5%)
Clinical chemistry	8/27 (29.6%)	5/19 (26.3%)	_^a^	_^a^
Clinical genetics	51/80 (63.8%)	117/148 (79.1%)	39/45 (86.7%)	45/50 (90.0%)
Clinical geriatrics	79/136 (58.1%)	169/240 (70.4%)	51/61 (83.6%)	123/140 (87.9%)
Pulmonology	83/443 (18.7%)	219/626 (35.0%)	102/193 (52.8%)	193/280 (68.9%)
Gastroenterologist	40/252 (15.9%)	141/493 (28.6%)	55/124 (44.0%)	156/254 (61.4%)
Medical microbiology	78/217 (35.9%)	120/284 (42.3%)	26/60 (43.3%)	61/98 (62.2%)
Neurosurgery	7/115 (6.1%)	20/159 (12.6%)	8/41 (19.5%)	14/44 (31.8%)
Neurology	163/730 (22.3%)	372/949 (39.2%)	145/269 (53.9%)	254/367 (69.2%)
Nuclear medicine	34/113 (30.1%)	70/188 (37.2%)	18/39 (46.2%)	24/51 (47.1%)
Gynaecology and obstetrics	301/893 (33.7%)	619/1082 (57.2%)	202/269 (75.1%)	310/371 (83.6%)
Ophthalmology	216/640 (33.7%)	322/723 (44.5%)	62/107 (57.9%)	99/168 (58.9%)
Orthopaedics	24/546 (4.4%)	90/798 (11.3%)	43/226 (19.0%)	76/263 (28.9%)
Pathology	2101/348 (29.0%)	194/440 (44.1%)	55/90 (61.1%)	106/147 (72.1%)
Plastic surgery	34/218 (15.6%)	92/329 (28.0%)	32/79 (40.5%)	65/111 (58.6%)
Psychiatry	899/2518 (35.7%)	1628/3432 (47.4%)	376/614 (61.2%)	536/744 (72.0%)
Radiology	142/913 (15.6%)	337/1218 (27.7%)	95/251 (37.8%)	193/407 (47.4%)
Radiotherapy	68/199 (34.2%)	163/299 (54.5%)	52/77 (67.5%)	79/106 (74.5%)
Rheumatology	73/196 (37.2%)	167/299 (52.4%)	60/87 (69.0%)	100/132 (75.8%)
Rehabilitation medicine	167/365 (45.8%)	323/531 (60.8%)	93/123 (75.6%)	131/156 (84.0%)
Urology	18/329 (5.5%)	95/441 (21.5%)	48/122 (39.3%)	81/145 (55.9%)
Sports medicine	0	46/139 (33.1%)	0	17/29 (58.6%)
Hospital medicine	0	0	0	28/173 (73.7%)
Emergency medicine	0	277/421 (65.8%)	4/4 (100.0%)	126/173 (72.8%)
**Total (all specialties)**	**4409/16405 (26.9%)**	**8699/21944 (39.7%)**	**2716/5233 (51.9%)**	**4477/6982 (64.1%)**

Data available on request by the Dutch Medical Registration Committee. https://www.knmg.nl/opleiding-herregistratie-carriere/rgs/registers.htm#Aantallen_geregistreerde_aios__(Registers)

^a^No data available.

## Appendix 2

Numbers and percentages of participants^a^ who completed the questionnaire in 2005 and 2015.

**Table:**

**Specialty**	**Participants 2005**			**Participants 2015**		
	**Total**	**Male**	**Female**	**Total**	**Male**	**Female**
Anaesthetics	147	65	82 (56%)	51	15	36 (70.6%)
Cardiology	104	67	37 (36%)	42	27	15 (35.7%)
Cardiothoracic surgery	11	9	2 (18%)	2	0	2 (100%)
Dermatology	40	7	33 (83%)	10	0	10 (100%)
General surgery	170	100	70 (41%)	71	42	29 (40.8%)
Internal medicine	291	104	187 (64%)	104	18	86 (82.7%)
Otorhinolaryngology	30	11	19 (63%)	24	9	15 (62.5%)
Paediatrics	162	34	128 (79%)	151	27	124 (82.1%)
Clinical chemistry	0	0	0	11	5	6 (54.5%)
Clinical genetics	24	5	19 (79%)	19	2	17 (89.5%)
Clinical geriatrics	28	3	25 (89%)	26	2	24 (92.3%)
Pulmonology	76	33	43 (57%)	45	12	33 (73.3%)
Gastroenterologist	45	21	24 (53%)	33	4	29 (87.9%)
Medical microbiology	25	11	14 (56%)	12	4	8 (66.7%)
Neurosurgery	10	7	3 (30%)	2	1	1 (50%)
Neurology	91	38	53 (58%)	49	9	40 (81.6%)
Nuclear medicine	6	4	2 (33%)	3	1	2 (66.7%)
Gynaecology and obstetrics	125	26	99 (79%)	84	14	70 (83.3%)
Ophthalmology	46	16	30 (65%)	14	5	9 (64.3%)
Orthopaedics	95	73	22 (23%)	42	31	11 (26.2%)
Pathology	30	9	21 (70%)	47	12	35 (74.5%)
Plastic surgery	32	16	16 (50%)	14	7	7 (50%)
Psychiatry	243	70	173 (71%)	87	18	69 (79.3%)
Radiology	78	38	40 (51%)	47	14	33 (70.2%)
Radiotherapy	45	9	36 (80%)	14	2	12 (85.7%)
Rheumatology	28	3	25 (89%)	14	0	14 (100%)
Rehabilitation medicine	81	21	60 (74%)	29	2	27 (93.1)
Urology	44	18	26 (59%)	56	23	33 (58.9%)
Sports medicine	0	0	0	6	3	3 (50%)
Hospital medicine	0	0	0	10	3	7 (70%)
Emergency medicine	0	0	0	29	6	23 (79.3%)
**Total (all specialties)**	**2107**	**818**	**1289 (61%)**	**1148**	**318**	**830 (72%)**

^a^Medical residents in PGMT

## Appendix 3

**Table:**

**Confirmatory factor analysis and reliability analysis for the UBOS for 2005 and 2015**			
		**2005**	**2015**
**Factor**	**Item**	**Standardized loading**	**Standardized loading**
Factor 1 (Emotional exhaustion; EE)	1	0.833	0.848
	2	0.792	0.796
	3	0.703	0.711
	5	0.689	0.752
	7	0.507	0.478
	8	0.876	0.865
	13	0.520	0.500
	18	0.788	0.801
Factor 2 (Depersonalisation; DP)	6	0.466	0.548
	11	0.810	0.714
	12	0.821	0.802
	14	0.474	0.469
	20	0.344	0.294
Factor 3 (Personal accomplishment; PA)	4	0.489	0.477
	9	0.592	0.575
	10	0.575	0.583
	15	0.696	0.734
	16	0.762	0.824
	17	0.632	0.620
	19	0.473	0.399
**Factor**	**Factor**	**Correlation**	**Correlation**
Factor 1 (EE)	Factor 2 (DP)	0.584	0.590
Factor 1 (EE)	Factor 3 (PA)	− 0.261	− 0.100
Factor 2 (DP)	Factor 3 (PA)	− 0.278	− 0.115
	**Model fit**		
	Comparative fit index (CFI)	0.904	0.897
	Tucker-Lewis Index (TLI)	0.891	0.883
	Root MSE Approx. (RMSEA)	0.067	0.070
**Scale**	**Estimator**	**2005**	**2015**
Factor 1 (EE)	Cronbach’s alpha	0.891	0.892
	McDonald’s omega	0.895	0.898
Factor 2 (DP)	Cronbach’s alpha	0.730	0.704
	McDonald’s omega	0.739	0.719
Factor 3 (PA)	Cronbach’s alpha	0.788	0.798
	McDonald’s omega	0.799	0.806

CFI, TLI and RMSEA indicate decent model fit. The only item with a somewhat lower standardized loading is item 20. However, Cronbach’s alpha and McDonald’s omega indicate good scale reliabilities, also for the scale that includes item 20 (DP). Therefore, we decided to retain item 20

**Table:**

**Confirmatory factor analysis and reliability analysis for the UWES for 2005 and 2015**			
Factor 1 (Vigour)	1	0.751	0.867
	2	0.805	0.885
	5	0.761	0.722
Factor 2 (Dedication)	3	0.825	0.899
	4	0.836	0.907
	7	0.702	0.743
Factor 3 (Absorption)	6	0.822	0.753
	8	0.646	0.759
	9	0.678	0.745
**Factor**	**Factor**	**Correlation**	**Correlation**
Factor 1 (Vigour)	Factor 2 (Dedication)	0.887	0.836
Factor 1 (Vigour)	Factor 3 (Absorption)	0.849	0.782
Factor 2 (Dedication)	Factor 3 (Absorption)	0.889	0.836
	**Model fit**		
	Comparative fit index (CFI)	0.959	0.941
	Tucker-Lewis Index (TLI)	0.938	0.912
	Root MSE Approx. (RMSEA)	0.090	0.123
**Scale**	**Estimator**	**2005**	**2015**
Factor 1 (Vigour)	Cronbach’s alpha	0.811	0.853
	McDonald’s omega	0.817	0.863
Factor 2 (Dedication)	Cronbach’s alpha	0.829	0.882
	McDonald’s omega	0.832	0.887
Factor 3 (Absorption)	Cronbach’s alpha	0.763	0.792
	McDonald’s omega	0.767	0.800

The CFI and TLI indicate good model fit, and the somewhat higher RMSEA value (lower is better) may be due to the very high correlations between the three factors. All standardized loadings are good and Cronbach’s alpha and McDonald’s omega indicate good scale reliabilities
